# TESTING THE GEROSCIENCE HYPOTHESIS

**DOI:** 10.1093/geroni/igae098.0319

**Published:** 2024-12-31

**Authors:** Monty Montano

**Affiliations:** Chair

## Abstract

This session explores the geroscience hypothesis through a series of cutting-edge talks examining the intersection of aging and chronic conditions, novel gerotherapeutics, and complex muscle, bone and adipose interactions.

